# Streaming the beautiful game: exploring big tech's growing presence in the soccer industry

**DOI:** 10.3389/fspor.2023.1156601

**Published:** 2023-06-29

**Authors:** Alexis Fakataulavelua, Markus Lang, Jérémy Moulard

**Affiliations:** Institute of Sport Sciences, University of Lausanne, Lausanne, Switzerland

**Keywords:** big tech, sports market, soccer industry, media rights market, technological development

## Abstract

This study investigates the evolving role of major technology corporations—namely, Amazon, Apple, Microsoft, Facebook, Netflix, and Google—in the sports industry, with a specific focus on soccer. By employing a qualitative content analysis of media reports, scientific literature, and annual reports from 2000 to 2021, the research scrutinizes the varying approaches and investments of these tech giants in the domain of sports. The findings classify these companies into three distinct categories: (1) those actively securing broadcast rights for major competitions and leagues (Google, Facebook, Amazon); (2) those primarily producing and disseminating soccer documentaries (Netflix); and (3) those not directly engaging in media rights but advancing the technological aspects of clubs and leagues (Apple and Microsoft). This study underscores the escalating significance of Big Tech in reshaping the sports media landscape and calls for further research to comprehend the broader implications of their presence in sports broadcasting and fan engagement.

## 1. Introduction

As we navigate an era marked by rapid technological progress and escalating global interconnectivity, the sports industry has seen a significant evolution, with Big Tech companies increasingly asserting their influence over the sports rights market. Titans like Amazon, Apple, Microsoft, Facebook, Netflix, and Google have harnessed their vast resources, technological expertise, and expansive user networks to challenge traditional broadcasters and transform the sports consumption landscape. As they stake their claims in this fiercely competitive market, these tech behemoths employ innovative strategies and cutting-edge technologies to secure broadcasting rights, engage fans, and broaden their global footprint. The impact of Big Tech's foray into the sports rights market is noteworthy, triggering a profound shift in the production, distribution, and consumption of sports content. Capitalizing on the surging demand for live and on-demand streaming services, these companies offer fans unprecedented access to their favorite sports and teams across various platforms and devices (1).

Consequently, the sports industry has become more dynamic, consumer-centric, and data-driven as Big Tech companies continue introducing new technologies and tools to enhance the viewer experience and cater to evolving audience preferences. Furthermore, the involvement of Big Tech firms in the sports rights market has intensified competition, driving up the value of sports rights and prompting traditional broadcasters to reevaluate their strategies and accelerate their digital transformation. As the battle for exclusive rights to major sports events rages on, the increased competition has fueled concerns over market monopolization, pricing, and accessibility, warranting further scrutiny from regulators and policymakers (2).

Our study aims to delve into the intricate relationship between Big Tech and sports, specifically concentrating on the soccer industry. We achieve this by examining the activities and strategic initiatives of six major tech companies: Amazon, Apple, Microsoft, Facebook, Netflix, and Google. The academic literature has, until now, largely overlooked the dynamics between these technology giants and their involvement in sports. Our study strives to partially fill this knowledge gap by providing insights into how these companies have shaped and continue to influence the sports landscape, particularly in soccer. We also seek to understand the motivations behind their engagement and their various roles as partners, content creators, and technology providers in the industry. By shedding light on these complex relationships, our research contributes to a better understanding of the contemporary sports ecosystem and the potential future directions for both the tech and sports sectors.

To conduct our research, we employed a qualitative content analysis of media reports, scientific literature, and annual reports published between 2000 and 2021. We identified 459 relevant sources within the selected timeframe to comprehensively understand the presence and activities of the six Big Tech companies in the soccer industry. We analyzed the data from these sources and organized it according to date, nature, subject, type of relationship, contract duration, and financial value. This approach allowed us to provide a detailed typological analysis of the actions of the six Big Tech companies in the soccer industry, which included four main categories and twelve sub-categories.

Our analysis enabled us to divide the six Big Tech companies into three groups based on their presence and activities in the soccer media rights market. The first group includes companies highly active in this market, such as Google, Facebook, and Amazon. They have been actively acquiring rights to broadcast major soccer competitions and leagues. The second group includes companies only interested in one type of content, such as Netflix, which has focused on producing and broadcasting documentaries about soccer. The third group contains companies that are not active in the media rights market but have a strong presence in the technological development of clubs and leagues, such as Apple and Microsoft. These companies have been instrumental in delivering innovative technological solutions and services tailored for soccer clubs and leagues. These solutions have significantly improved fan engagement and have streamlined various operational activities. Our analysis delves into the strategies, collaborations, and investments these Big Tech entities have undertaken in the soccer industry and how these actions influence the industry. The study underscores the escalating influence of Big Tech companies in the sports media ecosystem, thereby advocating for more comprehensive research to ascertain the future implications of their involvement in sports broadcasting and fan engagement.

The paper is structured in the following manner: Section 2 provides a background on the relationship between broadcasting and sports, including a brief history of sports broadcasting, an overview of the media market, and a brief examination of Big Tech's involvement in sports broadcasting. Section 3 explains the methodology used for the research, including the data collection and analysis techniques applied. Section 4 presents the study's results, including a typology of the six Big Tech companies based on their activities in the soccer industry. Section 5 concludes the paper, summarizing and discussing the main findings and suggesting areas for future research.

## 2. Background: broadcasting and sports

### 2.1. A brief history of sports broadcasting since the 1980s

Deregulation in the 1980s, which opened the market to new technologies and dismantled Europe's public and private free-view monopolies (3, 4), has thoroughly restructured the sports broadcasting market (5, 6). This deregulation has given owners more control over commercializing their rights (7) and transformed the media rights market (8, 9). Breaking up the television cartel has also significantly increased the number of media operators who can access different rights groups (10), leading to an exponential increase in competition for these rights (11–13). As a result, the pay television market has become the primary funder of spectator sports since the 1990s (14).

The growth of the soccer media rights market during the 2000s sparked intense competition among media operators (15, 16), particularly with the entry of telecom companies in several European countries, such as BT Group in England (7, 17). This influx of high-spending telecom companies intensified the competition between private and public operators and attracted the attention of Big Tech companies to the market (18).

The sports industry has witnessed a profound transformation in recent years, primarily in terms of consumption and distribution patterns. This change has mainly been propelled by technological advancements and the burgeoning influence of digital media (19). Consequently, the market has seen an array of disruptions, such as a slump in domestic broadcasting rights prices, the disintegration of media firms like Mediapro, championship suspensions due to the COVID-19 pandemic, and the ascendancy of over-the-top (OTT) streaming platforms (20).

The catalyst behind these upheavals lies in the evolving habits of consumers. As mobile devices and high-speed internet have become ubiquitous, sports enthusiasts gravitate more towards online platforms for enjoying their preferred games and events. OTT platforms have mainly gained traction as they enable viewers to circumvent traditional broadcast channels, accessing content directly *via* the internet. While this paradigm shift has paved the way for novel opportunities for content creators and broadcasters, it has also disrupted established business models, compelling companies to adjust to these new market realities.

However, the surge in OTT platforms isn't the sole disruptor in the sports industry. The downfall of Mediapro, a leading media company that procured the rights to broadcast Ligue 1 soccer matches in France, was a severe blow to the industry. This event underscored the inherent risks associated with aggressive bidding for broadcast rights and the complexities of monetizing these rights in a market that's becoming increasingly competitive and fragmented (21, 22).

### 2.2. Specificities and transition of the media market

The demand for a wide variety of programming types, encompassing live and non-live events, unencrypted and encrypted content, as well as diverse broadcasting methods such as terrestrial, cable, satellite, and digital, has led to a highly fragmented market (7, 23). This segmentation is further exacerbated by varying commercialization approaches, including free, subscription-based, and pay-per-view models (24). As a result, consumers seeking access to premium content often find themselves compelled to subscribe to multiple platforms to fulfill their diverse viewing preferences (25). The ramifications of this market fragmentation extend beyond consumers, impacting the strategies and operations of media industry players. Traditional broadcasters are compelled to adapt to the evolving landscape by diversifying their content offerings and embracing digital transformation to retain their audience base. Meanwhile, new entrants, such as Big Tech companies and streaming services, leverage their technological capabilities and deep pockets to secure exclusive rights and develop innovative viewing experiences for consumers.

This highly segmented market also presents challenges for sports leagues and organizations, as they must navigate complex negotiations and partnerships with multiple broadcasters and platforms to maximize their revenue and reach. Additionally, they must balance the need to maintain broad exposure for their sport while capitalizing on the lucrative opportunities presented by exclusive rights deals. Furthermore, the fragmented market has implications for regulators and policymakers, who must address concerns related to market concentration, consumer protection, and fair competition. For example, European law seeks to prevent monopolies by prohibiting the sale of rights to a single buyer, which is achieved by mandating that rights be shared by at least two operators (8, 26, 27). The European Commission encourages the division of rights into multiple packages and the utilization of open, transparent, and non-discriminatory bidding processes. This approach ensures a competitive market and safeguards consumer interests by promoting the accessibility and affordability of sports media content. As the media landscape continues to evolve, it is essential for regulators to stay abreast of industry developments and adopt policies to ensure a balanced and sustainable market environment.

In recent years, professional soccer clubs have sought to create value and establish direct relationships with fans by launching their TV channels and producing content (26). This strategy allows clubs to resist the dominance of traditional media companies and increase their presence in the global sports market. To make the most of these opportunities, rights holders have started exploring models that combine exclusive and non-exclusive content and content that can be accessed by paying extra (10). This approach recognizes that selling media rights exclusively to telecom operators can be inefficient and generate negative externalities (28). Evens et al. (7) suggested that rights holders should consider adopting a shared revenue model that allows new platforms to enter the market and increase competition. Some rights holders have begun exploring the option of offering multimedia platforms the possibility of buying non-exclusive licenses. Additionally, traditional media companies are encouraged to adapt and put digital technology at the heart of their commercial activities by forming partnerships with Big Tech companies (29).

In the early years, the potential impact of the internet on sports broadcasting was not fully recognized. However, it is now seen as a source of innovation, new markets, and additional revenue (30). In recent years, companies buying sports media rights have often included digital rights (31). However, these digital rights were often underutilized or considered secondary products (7). In many cases, companies acquired them only to deter emerging digital competitors such as Snapchat and Twitter. Clubs and leagues hesitated to embrace digital broadcasting due to the established relationships and high revenues from traditional broadcasters. The limited revenue from online channels at the time also discouraged the shift (32). As a result, digital and mobile rights had little impact on how soccer-related content was consumed.

### 2.3. Big tech's involvement in sports broadcasting

Big Tech refers to the world's largest information technology companies. The six companies examined in this paper—Amazon, Apple, Google (Alphabet), Facebook (Meta), Microsoft, and Netflix—had a combined revenue of $817 billion in 2018 (see Table 1), which represents 0.94% of global GDP in 2018.

**Table 1:** Summary of the Big tech companies’ business models.

Creation	Company	Main business model	Revenue 2018 (in B$)	Revenue 2021 (in B$)	Examples of acquisitions
1998	Google (Alphabet)	Advertising	136,8	257,6	YouTube, Android, reCAPTCHA, Waze
2004	Facebook (Meta)	Advertising	55,8	117,9	Instagram, WhatsApp, Oculus VR
1994	Amazon	E-commerce	232,8	469,8	IMDb, Twitch, Whole Foods Market
1997	Netflix	Subscription	15,8	29,6	The Roald Dahl Story Company, Next Games, Millarworld
1976	Apple	Hardware	265,5	365,8	Siri, Beats Electronic, Shazam
1975	Microsoft	Software	110,3	168,1	Skype, LinkedIn, Lionhead Studios

Sources: (33–38).

These companies and other tech companies, such as Twitter and Yahoo, began to enter the sports rights market by partnering with prestigious sports leagues (7, 18). For example, in 2016, Twitter paid the National Football League $10 million to broadcast ten matches through its Periscope app and signed a deal with Major League Baseball to broadcast games. Yahoo provided free live broadcasts of National Hockey League games the same year. In 2019, Google offered live coverage of 13 MLB matches during the second half of the regular season, Amazon secured a second lot of domestic rights to Roland Garros for 2021–2023, and Facebook signed an agreement to provide live coverage of the 2019 Masters Golf tournament in the Middle East (media rights are divided into two parts: domestic rights, sold to national television networks, and international rights, sold outside the national territory).

With billions of users, Big Tech companies are disrupting the traditional sports broadcasting industry. Some Big Tech companies are becoming major media outlets by offering over-the-top (OTT) media services alongside specialized companies like DAZN (39). They are called the “Netflix of sport” (2). With fully globalized operations in various markets, these companies have significant business growth resources. Their recent interest in sports, along with the emergence of new distribution methods such as B2B2C and DTC, in addition to traditional B2B distribution networks, is transforming the media rights landscape and forcing the sports industry to re-evaluate its economic model (40).

## 3. Methodology

This study utilizes a qualitative content analysis of media reports, scientific literature, and annual reports to gain a deeper understanding of Big Tech companies' involvement in the soccer industry. Our research focuses on six major companies: Amazon, Apple, Microsoft, Facebook, Netflix, and Google, chosen based on the following five cumulative criteria: (a) an internet-related business model; (b) a company not initially associated with media production; (c) an annual revenue exceeding $10 billion between 2018 and 2021; (d) a company that expressed interest in the soccer industry before 2021; (e) a company listed on the NASDAQ-100 stock market index. To substantiate the decision to set the limit at $10 billion, we analyzed the revenues of the 103 companies listed on the NASDAQ-100 stock market index. Specifically, we calculated the median revenue figures for 2018 and 2021, which were found to be $9.2 billion and $10.95 billion, respectively. Based on these results, we decided to set the limit at $10 billion.

Qualitative content analysis is a method that interprets and describes the topics and themes present in communication content, aiming to identify central consistencies and meanings (41, 42). This method enabled us to systematically categorize and organize the content into themes, categories, and patterns, thus offering a comprehensive understanding of Big Tech companies' activities within the soccer industry. Given our research objective of exploring the role of Big Tech in the soccer industry, this method proved to be especially appropriate. We identified several key themes and trends in Big Tech companies' soccer industry activities through qualitative content analysis. Our analysis also highlighted the significance of partnerships between Big Tech companies and traditional broadcasters, as well as the potential impact of emerging technologies on the future of soccer media.

The data collection process involved several stages. Initially, we identified articles by searching databases using search engines (e.g., Google Scholar, Google, Bing) and specialized websites in various languages (e.g., sportspromedia.com, broadbandtvnews.com, sportstrategies.com, digital-football.com, calcioefinanza.it, ecofoot.fr, sportbuzzbusiness.fr, sportsmarketing.fr, mediasportif.fr). We employed multiple keywords (e.g., big tech* OR gafa* OR fangam* OR gafam* OR faang* OR big five* AND sport* AND football*, as well as combinations such as the name of the big tech company* AND sport* AND football* AND/OR media rights*) for the period from January 1, 2000, to December 31, 2021.

Our initial search yielded 474 results. After eliminating duplicates and verifying articles, we identified 459 relevant sources within the selected timeframe. The distribution of sources was as follows: 39% related to Amazon, 23.25% to Facebook, 17.5% to Google, 9.25% to Microsoft, 7% to Apple, and 4% to Netflix. We analyzed the data and organized it according to date, nature, subject, type of relationship, contract duration, and financial value. Finally, we manually transcribed the collected data into a database.

**Figure 1:**
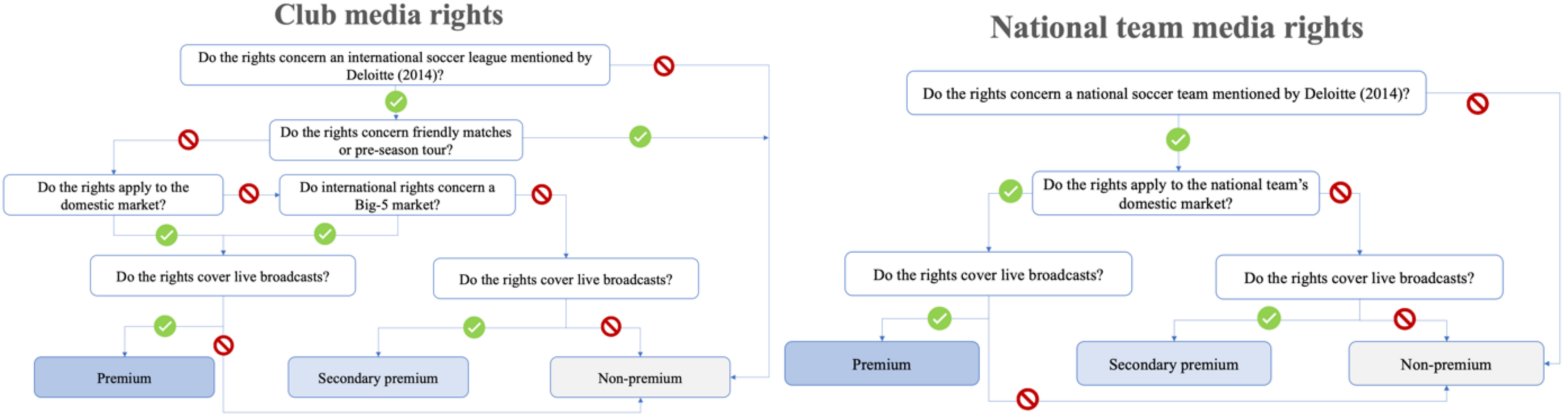
Categorization of media rights. Source: Own creation.

The typology derived from our analysis comprises four primary categories of actions (media rights purchaser, broadcast rights partner, media technology partner, sports technology partner), each encompassing several sub-categories. We generated a comprehensive overview of their involvement in the soccer industry by assigning each company's activities to the corresponding categories and sub-categories. The media rights purchaser category in our typology includes three sub-categories of rights: premium rights, secondary/alternative premium rights, and non-premium rights. However, determining and allocating rights to these sub-categories raises the question of what constitutes each type of right, which has not been widely discussed in the literature. Therefore, each player in the media rights market assigns a subjective value to each right and has its definition of what constitutes a premium right (43). To address this issue, we used financial reports (e.g., PWC's annual Sports Survey) and an interview with a media rights specialist to develop a working definition of premium, secondary/alternative premium, and non-premium rights that we applied consistently across our analyses (see Figure 1).

## 4. Overview of Big tech's involvement in soccer

Our analyses enabled us to divide the Big Tech companies into three groups based on their presence and activities in the soccer media rights market: a group of companies that are highly active in this market (Google, Facebook, Amazon), a group that is interested only in one type of content—documentaries (Netflix), and a group that is not active in the media rights market but has a strong presence in the technological development of clubs and leagues (Apple, Microsoft). The following section provides an overview of these companies, their investments, their involvement in soccer, and their motivations.

### 4.1. Google, Facebook, and Amazon: the most active Big tech companies in the media rights market

#### 4.1.1. Google

**Figure 2:**
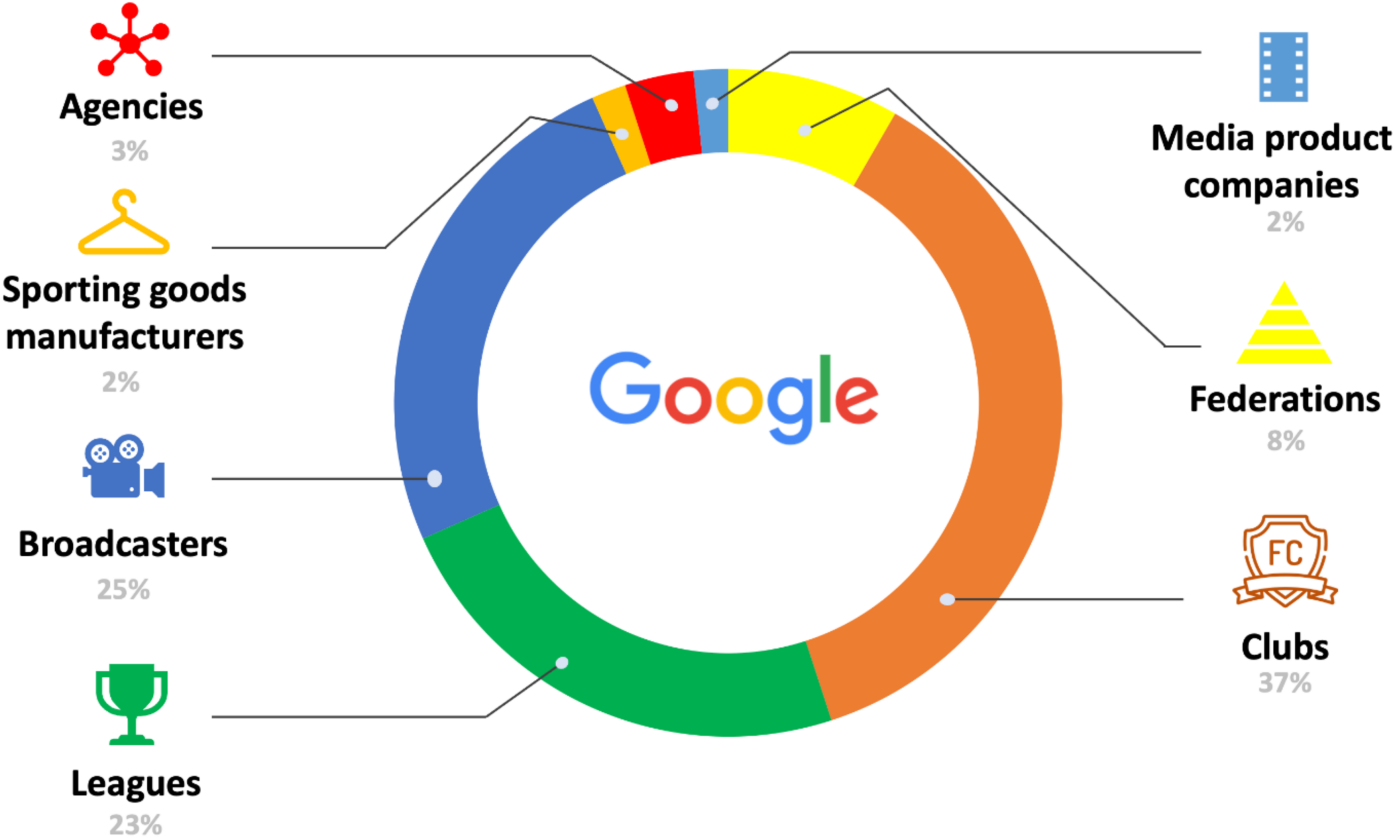
Google's relations with the soccer industry (in %). Source: Own creation.

Google was one of the first Big Tech companies to broadcast soccer matches, starting in 2011 with live coverage of games from various confederations and leagues. The company exclusively broadcasts sports content through its subsidiary YouTube and abandoned tests of live broadcasts on Google + and Hangout in 2018 and 2020, respectively. Additionally, it has explored technological innovations through partnerships with clubs and leagues, such as its collaboration with AS Roma in 2014, where the club's coach wore Google Glasses, allowing viewers to see events from the coach's perspective. Google mainly partners with clubs and media outlets (see Figure 2) that aim to enhance their media presence and revenues.

Google made history as the first Big Tech company to acquire broadcasting rights to a professional soccer league when YouTube obtained the non-live rights for France's League 1 in 2012. YouTube renewed the contract, which includes the rights to show goals, match summaries, and highlights, in 2017. Furthermore, Google has acquired live premium rights, such as in the United States, through its YouTube TV service. The cost of an annual subscription to YouTube TV has increased from $35 at launch in 2017 to $65 in 2023.

YouTube initially provided free access to all content, generating income through advertising revenues it shared with content producers. However, this model did not allow for direct payment from consumers. To address this, YouTube began testing alternative consumption modes to monetize its content. In 2015, MediaPro and La Liga signed an agreement with YouTube to broadcast Spain's Copa del Rey in 20 international territories, charging €19.99 for all matches or €4.99 for individual games. By the end of 2019, Liverpool FC became the first European club to ask fans to pay a subscription (£0.99 per month) for exclusive non-live content on YouTube.

In 2020, Russia's Premier League (RPL) offered fans in territories outside Russia the possibility to access live broadcasts of the rest of the season's games, with two subscription rates: $2.99 per month for limited access and $4.99 for all the matches. However, these alternative consumption modes can raise contractual issues with rights already sold to broadcasters and advertisers. YouTube's Head of Sport for the EMEA region, Rob Pilgrim, stated, “If you have sponsorship deals in place and you're worried about potential conflicts, then you can block those competitors from advertising on your content… We're really trying to enhance the monetization tools we have, and last year we paid US$30 billion to YouTube partners. That's a significant number and growing every year” (44).

Live content has become crucial for Google, with 48.5% of its activities involving live rights partnerships. Rather than purchasing rights, YouTube incentivizes rights owners and broadcasters to offer free live content in exchange for a share of ad revenues, promoting the “YouTube-ization” of sports. Rob Pilgrim explains, “We don't believe buying sports rights will add much value for us…We have no plans [to invest]” (45). These secondary premium or non-premium rights follow a consistent model: free, global streaming to growing markets for easy audience expansion. Tomos Grace, YouTube's Head of Sports for EMEA, observes that BT and Sky see YouTube as a marketing tool (46). In theory, globally available free products act as loss leaders, attracting clients who may later subscribe to premium offerings.

YouTube is an ideal platform for engaging a global, young, and female audience. For example, Tomos Grace emphasizes that the YouTube membership of the Liverpool FC channel represents a new opportunity for sports organizations to reach a global audience (47). In addition, broadcasting UEFA Youth League and National Women's Soccer League (NWSL) matches also cater to younger and female viewers. The recent DAZN and YouTube four-year agreement with UEFA for free broadcasting of the UEFA Women's Champions League highlights their aim to attract more female consumers (48).

By making its platform available to its partners, YouTube can optimize its revenues by taking a percentage of advertising receipts and subscription fees while gaining access to vast data. Google's algorithms provide detailed profiles (location, type, duration, comments, variety of content watched) for targeting ads while providing event broadcasters with new opportunities. For example, the RPL's YouTube subscription offer enabled the league to evaluate its attractiveness outside Russia and test subscription rates. In 2019, Sky Sports' decision to broadcast English Premier League highlights on YouTube resulted in the channel adding more than 200,000 subscribers in a month (46). Popular non-live content, such as match highlights, generates more detailed data on consumption behaviors and provides an optimal window for advertising revenues in the long term. According to Rob Pilgrim, “Google is the largest search engine in the world, and YouTube is the second largest. People expect highlights when they come to the site. People come to YouTube to search after major sporting events, and there is a 97% chance you will find the moment on YouTube legitimately” (44).

In summary, these findings highlight traditional broadcasters' attempts to offset declining subscriber numbers by forming alliances with YouTube for short, non-live formats, coupled with free live broadcasts to restimulate their audience and attract new viewers to their premium products. At the same time, clubs and leagues are exploring ways to directly monetize sub-communities of fans by providing various forms of access, including through subscription services, to rights and content that are currently under-exploited. They provide simple, effective, and global offers to reach new consumer markets. Based initially on free-to-view content and advertising revenues, YouTube has moved to a mixed model (free, premium) that gives content producers various broadcasting and monetization options. Free distribution, existing multi-year contracts, and the legal protections provided by national markets limit Google to a complementary relationship with rights holders. Therefore, the internet giant is becoming a major player in sports entertainment by capitalizing on the importance of non-live products built around live events, as displayed in Panel (a) of Google's typology diagram in Figure 5 below.

#### 4.1.2. Facebook

An English FA Cup match between two ninth-division clubs, far from the European elite, in 2011 was the first soccer game to be broadcast live on Facebook (On October 28, 2021, Facebook, Inc., the parent company of the social media giant, rebranded itself as Meta Platforms, Inc.). Since then, leagues and clubs have put an increasing amount of content on Facebook, which has become one of the most active and aggressive actors in the soccer rights market. To take advantage of the digital transformation, in 2016, Facebook paid substantial sums to famous clubs and players, including FC Barcelona ($1 million), Real Madrid ($917,000), and Iker Casillas ($211,000), to produce video streams for Facebook Live. Facebook's aim in persuading these actors to put more content on its platform was to create a buzz among their large communities of followers and build demand for more sports content. Facebook's initiative resulted in clubs of all sizes putting large amounts of live content on its platform. Peter Hutton, Facebook's Head of Sports, explained the company's reasoning: “You want people to be active viewers. We estimated that somebody who engages with that content watches four or five times longer than someone who sits as a passive consumer” (49).

**Figure 3:**
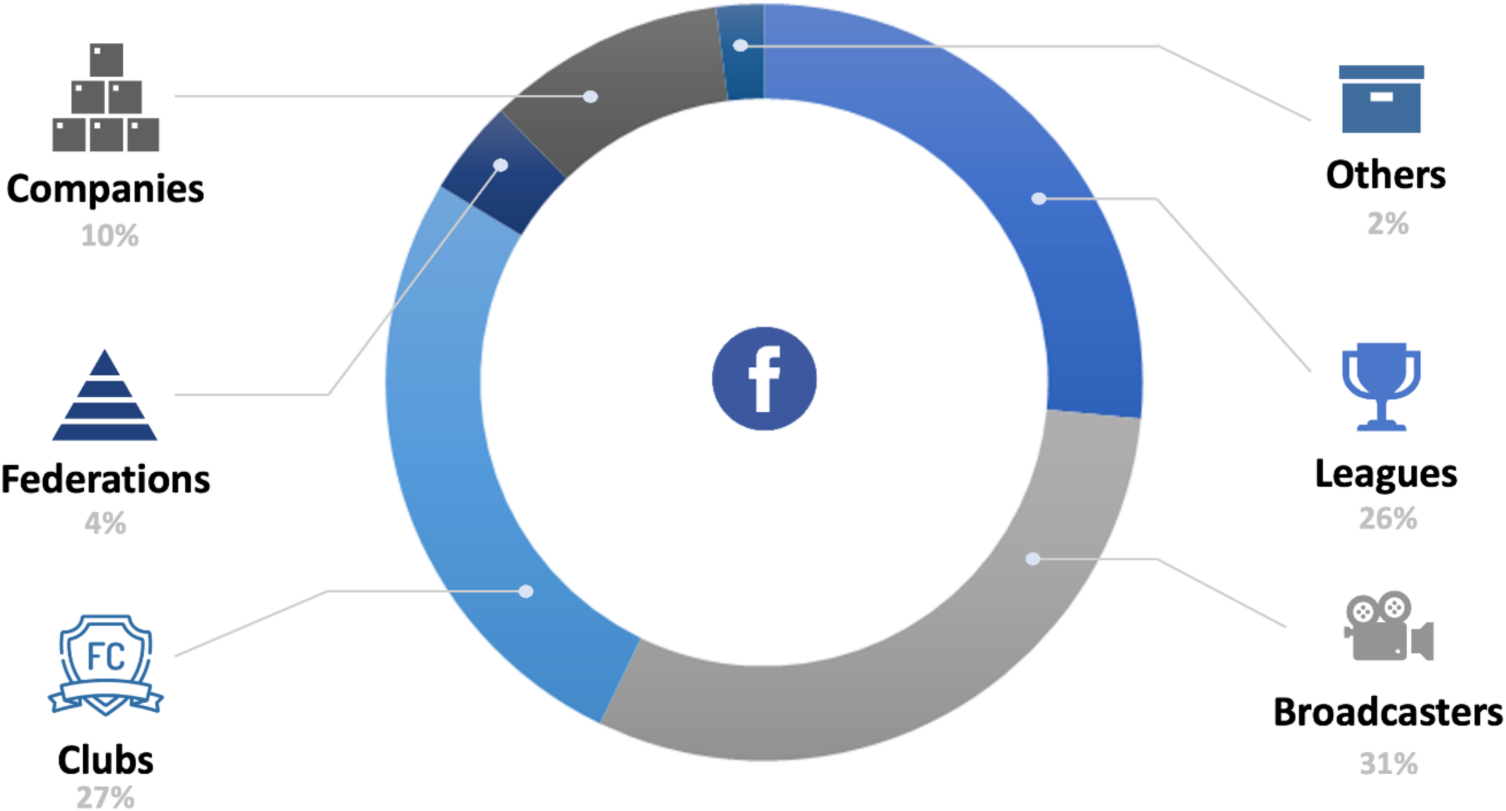
Facebook's relations with the soccer industry (in %). Source: Own creation.

To acquire production and broadcasting skills, Facebook collaborated closely with leagues, media-rights agencies, multinationals, and traditional broadcasters to distribute live content (see Figure 3). Additionally, Facebook is positioning itself as a partner platform to assist clubs and leagues with digital transformation. Its commercial partnerships with companies such as TicketMaster (to sell tickets online *via* Facebook), acquisitions of sports media start-ups, and recruitment of sports media rights specialists demonstrate its desire to expand into the sports media field. Facebook uses content such as a documentary about Real Madrid, produced in collaboration with the club and GoPro and shown exclusively on Facebook Watch, as a loss leader to generate interest in its products. Facebook buys very little of the live content on its platform, preferring to follow a platform economy model in which it pays most of the advertising revenues it receives to the broadcaster. According to Peter Hutton, “95 to 96% of live sport on Facebook is not paid for by the company” (49). Given Facebook's global reach, with over a billion daily users worldwide and a usage rate that soared during the Covid-19 crisis, the audience for live streams is enormous and, therefore, monetizable *via* advertising.

Like Google, in 2021, Facebook launched another pay-per-view function that allows content producers to monetize their audiences directly. Rob Shaw, Facebook's Director of Sports Media and League Partnerships, explains: “I think pay-per-view is by no means on any verge of extinction. This is something that helps breathe new life into it. People are willing to pay to experience a moment” (50). With this new offer, minor rights holders can increase their profits but at the risk of reducing their audience. In contrast, primary rights holders must face the complexity of exclusive contracts with subscription television networks and geolocation challenges regarding broadcasting rights. The transformation of the media market is pushing actors to monetize their large communities' access to live and non-live elements. Clubs such as Real Madrid and FC Rapid 1923 have used Facebook's fee-paying direct-to-consumer (DTC) function Fan Subscriptions, launched in 2018, to engage their fans and increase revenues. Facebook has responded to this success by expanding its royalty rate to 30% of revenues obtained from all new subscribers since January 2020.

Since 2018, Facebook has actively acquired secondary premium rights for broadcasting sports events. Some notable acquisitions include the rights to broadcast the English Premier League in certain Asian countries until 2022 for $264 million, the rights to air 380 Liga matches in the Indian market until 2022, the rights to broadcast 32 UEFA Champions League and UEFA Europa League matches in Latin America until 2021, and the rights to air 46 Copa Libertadores matches in Latin America and “behind-the-scenes” content from the Copa America in 2019. These acquisitions help gather data on fan behavior and consumption habits. For example, Latin American agreements revealed that over 8.8 million viewers, with 71% under 35 years old, tuned in, generating 1.3 million comments and 10 million reactions on Facebook's UEFA Champions League page. Peter Hutton emphasizes the importance of learning from sports coverage and avoiding big gambles that might disappoint fans (49).

Despite initial success in acquiring sports broadcasting rights, Facebook faced setbacks, including losing contracts for the English Premier League in Asia and exclusive Copa Libertadores matches, which led to reduced short-term investment in soccer and sports. Peter Hutton emphasized the importance of pacing and prioritizing long-term success. Facebook's decision not to renew contracts with UEFA and La Liga aligns with Hutton's statement, “I am certainly not expecting any huge investments in sports rights in the near future” (49). Rob Shaw adds that traditional media rights deals don't align with their current video business model (51).

**Figure 4:**
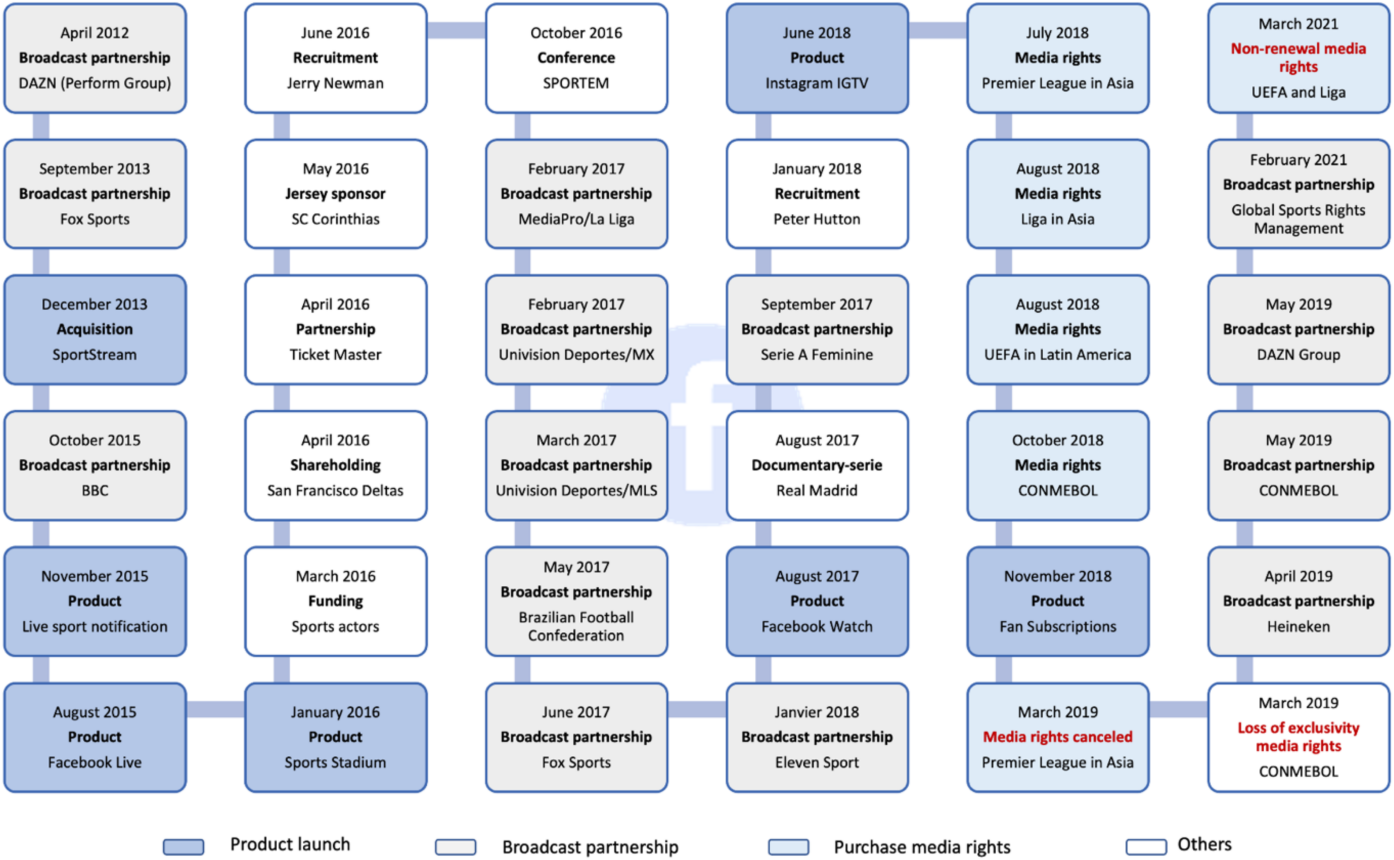
Chronology of Facebook's actions in sport and in soccer. Source: Own creation.

In summary, Facebook has been working towards providing live coverage of sports events since 2011, as illustrated in Figure 4.

**Figure 5:**
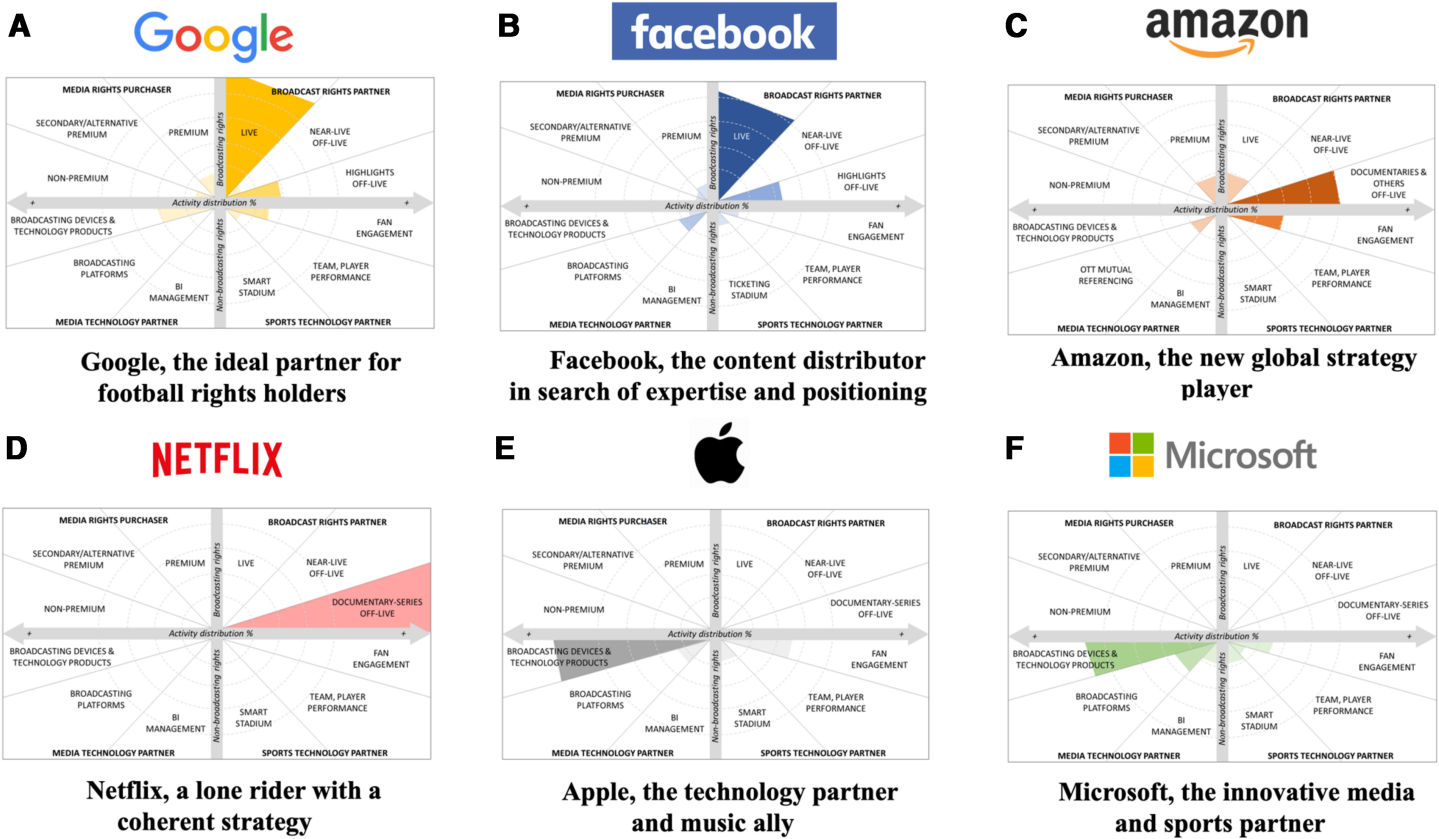
Presence of Big tech companies in professional soccer. Source: Own creation.

The company has gained expertise through acquisitions, product launches, hiring specialists, and partnerships for broadcasting rights. They have also developed a variety of technological solutions such as Facebook Live, Facebook Watch, live sports notifications, Bot Messenger, 360 degree videos, Sports Stadium, Fan Subscriptions, Oculus headsets, etc., to test the market and find the best strategy for leveraging its social and economic potential. For example, the realization of how strong the competition was to acquire the rights to broadcast events in secondary international markets combined with the fact that its economic model would not allow it to charge for access to this content, Facebook decided to reorient its strategy. This trial-and-error process has allowed the company to gather valuable insights into fan consumption behavior and preferences. Despite temporarily pausing its aspirations to provide live content, Facebook remains a valuable broadcasting partner for independent sports event organizers of all sizes as an alternative to traditional models. Facebook's typology diagram is illustrated in Panel (b) of Figure 5 below.

#### 4.1.3. Amazon

Amazon's entry into the sports industry was unexpected, with its first move being the sponsorship of Polish soccer club Śląsk Wrocław's shirts for two matches in 2015. This move aligned with Amazon's goal of opening logistics centers in Wrocław and creating jobs while contributing to the local community. Since then, Amazon has built a media empire around its Prime Video service, which provides access to a wide range of series, films, and sports-related documentaries to its over 200 million subscribers. The service offers live events and non-live documentaries that complement each other, forming a powerful traffic builder for the Prime platform.

Amazon's strategy includes integrating its technology products into its broadcasting service. For example, it has entered partnerships with clubs and media outlets to distribute Amazon's intelligent personal assistant, Alexa, which can create new sports consumption experiences that include voice and visual control of the content displayed (match statistics, analysis, live comments, etc.). In addition, Amazon has not forgotten its core business of online shopping. In 2018, it signed contracts with several clubs to open official shops on its online shopping website. By outsourcing their sales process to Amazon, clubs can eliminate the need to manage orders and distribution, saving themselves a great deal of hassle. However, it is essential to note that Amazon typically takes a percentage (around 15%) from the sales, which may decrease the clubs' profit margins. Despite this, Amazon's vast global distribution network provides clubs with an excellent opportunity to promote their brand, reach new international fans, and significantly increase their sales volumes, which can help to offset the loss of margin. These agreements may open new opportunities, as shown by Amazon's decision to become SSC Napoli's left arm sleeve sponsor for 2021–2023 after the club had opened an official shop, supplied by Kappa, on Amazon in 2017.

Amazon has been a recent player in the soccer market. The company first showed its interest in the market in 2016 and 2017 by acquiring internet, mobile, and audio rights for Germany's Bundesliga 1 and 2 matches and buying the rights to show video highlights of MLS games *via* its subsidiary Twitch. In 2018, Amazon made a groundbreaking move by becoming the first major tech company to obtain domestic rights to broadcast a top-tier European championship: the English Premier League. With a substantial investment of £90 million, Amazon secured the rights to broadcast 20 Premier League matches each season for three consecutive seasons, spanning from 2019/20 to 2021/22. This marked an unprecedented move for a streaming service, offering a level of coverage previously unheard of and effectively transforming the landscape of sports broadcasting. Amazon Prime subscribers in the UK were given access to these matches, enriching their membership with high-profile sports content in addition to the existing benefits. Amazon further amplified its reach by making these matches available on Twitch, a platform primarily known for video game live-streaming. This strategic move enabled Amazon to tap into a broader, potentially younger demographic, leveraging the popularity of Twitch to introduce a new generation to football.

With an impressive 579 billion minutes viewed in 2019 and 55% of its audience within the 18–34 age bracket, Twitch caters to a youthful gaming audience. Recognizing the demand for sports content, Twitch strives to establish a vibrant ecosystem. Sports organizations are increasingly collaborating with the platform, as demonstrated by the 2020 agreement with the National Women's Soccer League (NWSL), which provided exclusive international rights and free access to 24 matches. The unique, personalized, and interactive nature of sports streaming incorporates elements of “gamification,” heightening viewer engagement and monetization opportunities. As articulated by Amazon's Marie Donoghue, the company aims to establish a “big tent” for fans, offering them a choice of consuming content either on Prime Video or Twitch (52).

Amazon also capitalized on the financial impact of Covid-19 on traditional broadcasters, taking over the final matches of the 2020 Bundesliga season following Eurosport's withdrawal and buying the rights to 80% of France's Ligue 1 matches for €259 million, three times less than the original sale price, when MediaPro canceled its contract. In addition, Amazon is a preferred live-broadcast partner for broadcasters and clubs, particularly for local minor rights. Additionally, it is expanding its presence through agreements to make broadcasters', leagues', and clubs' OTT channels available on Prime Video and to make Prime Video available through other media companies' decoders.

Amazon Web Services (AWS), the company's cloud computing division, is a global leader, ahead of competitors Google and Microsoft. It has been a significant source of Amazon's global operating profit, accounting for 60% since 2014. AWS offers a wide range of cloud services, including machine learning, storage, data computation, AI, VR, AR, IoT, and media management. Several organizations in the sports industry, such as DAZN and Arsenal FC, have signed contracts with AWS for cloud services and broadcasting. However, AWS has a particularly close relationship with the Bundesliga, working together since 2020 to develop new statistics and predictions for fans and to create a personalized content experience. AWS's machine learning system is based on a combination of live data, historical data of over 10,000 Bundesliga matches, and over 150,000 h of videos, which are tagged to facilitate the retrieval of information such as players, teams, and the broadcasting times of sponsors (53).

Amazon also offers fans x-Ray functions, which allow them to personalize their live match experience. Marie Donoghue emphasizes the importance of customization for fans, who may have different preferences for statistics, commentary, and other features (52). AWS also uses sports as a testing ground for its AI facial recognition technology and for exploring automatic learning and hybrid reality. Amazon has focused on prestigious and growing leagues to capitalize on fan passion and measure adoption and engagement rates for future investments. Marie Donoghue explains that the company studies the desires of its members before entering a new market and chooses to enter countries with the largest and most followed events (54). Additionally, Amazon starts with the client, the territory, and the country and evaluates which sports are most attractive to these audiences. She further states sports are largely inherently local, and Amazon uses sports to drive engagement and focus to its overall Prime Video offering, so fans don't just come in and watch the Premier League (52).

In summary, Amazon's subscription-based model aligns well with the sale of soccer media rights. The company aims to create an entire consumption ecosystem around soccer, including its cloud services, e-commerce platforms, and Twitch. The company plans to achieve this by broadcasting matches live during the holiday period, using Alexa to enhance the viewing experience, promoting online product sales, broadcasting documentaries, and partnering with traditional broadcasters to gather data on consumption behaviors. Amazon's growing interest in the sports industry has led it to target multiple sub-markets, such as media rights, e-commerce, video games, sports betting, and cloud computing, which intersect within the realm of entertainment and play a mutual role in driving traffic. Amazon's focus on understanding fan and player behaviors suggests that the company aims to control the past, present, and future by curtailing the unpredictability of sports for commercial purposes. Amazon's machine learning expertise could allow it to become a highly influential but shadowy intermediary between the various sports markets (media, transfer, betting, sporting goods), federations, and clubs, giving a significant degree of control over soccer and the whole sports ecosystem. Panel (c) of Figure 5 below visualizes the typology diagram of Amazon.

### 4.2. Netflix: the non-live media rights partner

Despite being frequently mentioned in calls for tenders from sports rights holders, Netflix's interest in sports is limited to documentaries. It has no plans to move into live content with value for only 90 min. According to Maria Ferreras, Netflix's Global Head of Partnerships, the company does not see how it can offer something different from a traditional television broadcaster regarding live sports (55). The intense competition between new online actors and conventional television networks to obtain premium live media rights may also be a reason for Netflix's reluctance to invest in this market.

Netflix's economic model, centered around a diverse catalog of sports content accessible on-demand, is exceptionally well-suited for creating in-depth documentaries that offer a comprehensive view of a club or player's season or career. These engaging documentaries, combined with the platform's extensive global reach, enable the American multinational corporation to generate interest in new competitions and reinvigorate diminishing events, drawing in a younger, more diverse, and international audience. A prime example of this is the first season of the “Drive to Survive” series, which is focused on Formula 1 racing. This captivating documentary series contributed to a resurgence in the competition's popularity, which had been waning in recent years. In 2019, the series attracted more than 5.1 million viewers in the UK alone, giving the championship a fresh, modern appeal that appealed to a broader audience. This innovative approach serves as an invaluable template for clubs or leagues aiming to boost their popularity or revitalize their brand image. By harnessing the potential of captivating storytelling and the extensive distribution network offered by platforms like Netflix, sports organizations can effectively engage both existing and prospective fans, igniting a renewed sense of enthusiasm and commitment for their respective events or leagues.

This model also empowers Netflix to enhance its market penetration and attract new subscribers by targeting highly globalized clubs and players that hold considerable added value in regions where soccer is deeply ingrained in the culture and garners substantial media attention. Maria Ferreras has noted that the company is eager to continue pursuing partnerships, as they believe such collaborations drive growth, promote dynamism, and deliver an exceptional consumer experience (55). In a strategic move to increase brand awareness, Netflix gifted subscriptions to 28 professional soccer players in 2017, introducing them to the platform. In exchange, the players were asked to share photos of themselves enjoying Netflix series on social media. Furthermore, in 2020, Netflix formed a partnership with the Mediapro-owned channel Téléfoot for Ligue 1 coverage, enabling them to provide a combined “Netflix + Téléfoot” subscription package for €29.90 per month. This alliance showcases Netflix's commitment to offering its viewers unique, comprehensive sports content.

In summary, Netflix's foray into the sports domain primarily focuses on generating non-live content for its subscribers, as illustrated in Panel (d) of Figure 5 below. Sports rights owners stand to gain significantly from this type of content, enabling them to weave captivating narratives around live matches, modernize and bolster their international brand image, and draw in new fans from across the globe. Moreover, this engaging content is a gateway to direct these fans toward the primary product—live games. By delving into the realm of sports documentaries and non-live programming, Netflix effectively taps into a new market, offering its audience an immersive experience that goes beyond the live matches themselves. This unique approach provides added value to subscribers and fosters a more profound connection between fans and their favorite sports teams, clubs, or players. Consequently, it enables the potential for increased viewership and interest in live games, further solidifying the bond between sports organizations and their global fan base.

### 4.3. Apple and microsoft: technology partners but inactive in the media rights market

The final group in this typology contains Apple and Microsoft, which have no presence in the media right market, neither for live content nor non-live content. However, they have entered the soccer market in other sectors, notably through technology partnerships with leagues, clubs, and players.

#### 4.3.1. Apple

Despite circulating rumors in 2012 suggesting Apple's potential interest in acquiring the media rights to the Premier League, the company never engaged in any negotiations for such a transaction. Yet, in a surprising move in 2016, Apple turned its attention to the American soccer scene, becoming a shareholder in the newly formed San Francisco Deltas franchise. This venture saw Apple join ranks with other tech powerhouses like Twitter, Facebook, Google, and PayPal. Apple also promotes its Beats Electronics earbuds and speakers through official supplier agreements with major clubs and well-known players with millions of social media followers. Additionally, it uses its music streaming platform, Apple Music, to cross-promote its products and places its products directly with fans through agreements with clubs such as AS Roma and Bayern Munich, under which players share their musical tastes with fans on Apple Music.

Apple promotes new digital payment methods by encouraging fans to use their iPhones' Apple Wallet and Apple Pay apps to pay for stadium purchases. The company also works with clubs on launching apps that give fans access to various types of club content.

Apple's presence as a club sponsor is limited. It has only had shirt-sponsorship contracts with a few Brazilian soccer clubs between 2016 and 2018, all of which were negotiated through its Brazilian retail sales network, iPlace. Like Amazon, these contracts allowed the clubs to sell tie-in products through iPlace and allowed Apple to promote and anchor its sales network.

In sum, the soccer industry represents a significant pool of consumers and a powerful platform for Apple's products. To continue its international growth, the company aims to embed its brand in parallel worlds and enter collaborations to entrench its core products. Modern clubs with high added value, solid cultural roots in soccer, and star players with large social-media followings are notable targets. As Apple develops Apple TV, it is likely to expand its involvement in the sports industry, as hinted by former Amazon executive James DeLorenzo's recruitment to head Apple TV's sports section. This move may signify that Apple plans to enter the soccer media rights market. The typology diagram of Apple's presence and activities in the soccer industry are displayed in Panel (e) of Figure 5 below.

In June 2022, Apple and Major League Soccer (MLS) announced a global broadcast partnership for ten years, beginning with the 2023 MLS season. As part of the $2.5 billion agreement, Apple will broadcast all regular season MLS matches and Leagues Cup games on the Apple TV platform, accessible *via* a streaming service within the MLS app. In exchange, MLS will receive a minimum of $250 million per season from Apple, which marks a significant increase compared to the league's previous agreements with Fox, ESPN, and Univision, which averaged $90 million per season over eight years. Since the agreement was reached after our data cutoff on December 31, 2021, we could not incorporate it formally into our analysis.

#### 4.3.2. Microsoft

Microsoft has been involved in soccer since 2005 and aims to maintain solid technological ties with leading European leagues, such as La Liga, and clubs like Bayern Munich and Real Madrid. Like Apple, Microsoft uses technology partnerships to embed its products, primarily Xbox, Surface, and Windows. In 2021, La Liga extended its partnership with Microsoft to develop new technological solutions around OTT, AR, and VR. The French “Ligue de Football Professionnel,” the governing body that runs the major professional football leagues in France, also partnered with Microsoft and Ooyala in 2019 to launch an OTT channel.

In addition, Microsoft helps clubs to increase their media visibility in new markets and internationalize their appeal by signing deals to provide exposure in new regions. In 2015, Bayern Munich signed an exclusive agreement to increase the club's coverage on the MSN portal in North America. Similarly, Spain's La Liga extended its partnership with Microsoft in 2016 to expand its international visibility. Chris Capossela, Microsoft's Chief Marketing Officer, stated, “For us, that is in some ways the perfect marriage where you have a sports club that has a good fanbase and a great brand and is looking for a technology partner” (56).

Microsoft, founded in Washington State, has a strong presence and history in Seattle, particularly with its sports clubs. From 2008 to 2018, Microsoft Xbox was a shirt sponsor for the Seattle Sounders FC men's soccer club, and in 2019, several Microsoft staff became part of the ownership group. Additionally, Microsoft sponsored the OL Reign women's soccer club in Seattle in 2016 and 2017, implementing a partnership that utilized Microsoft technology for centralized data access and real-time analysis to improve match preparation and prevent injuries. Microsoft opened the Global Sports Innovation Center in Madrid in 2015 to promote innovation in the sports industry and launched a technology incubator product with La Liga in 2019.

In sum, Microsoft aims to be a technological partner for the sports industry rather than simply purchasing rights. Like Apple, it seeks to expand the use of its technology products and services in high-value soccer brands and increase the appeal of its products to fans. Microsoft assists its partners in modernizing their facilities and gaining insights into fan behavior through data. Its expertise and products in OTT, AR (HoloLens), and VR (Windows Mixed Reality) will further engage fans in a mixed virtual world (Microsoft Mesh). Instead of buying media rights directly, Microsoft prefers to form alliances with rights owners to provide media distribution tools. Like other Big Tech companies, Microsoft uses sports as a testing ground for developing and refining new technologies and algorithms that can be applied to other markets. Panel (f) of Figure 5 below displays the typology diagram of Microsoft's presence and activities in the soccer industry.

### 4.4. Summary of the results

This study aimed to examine the presence and activities of Big Tech companies in the soccer industry. Figure 5 visually represents the typology diagram, effectively demonstrating the various roles and relationships that Big Tech companies have established within the soccer industry. This classification system offers valuable insights and facilitates understanding the complex interactions and partnerships between Big Tech companies and the soccer industry. The upper section of the typology diagram shows a company's presence and activities in the media rights market, either as a “media rights purchaser” or as a “broadcast rights partner”; the lower section shows a company's presence and activities in the non-broadcasting rights market, either as a “media technology partner” or as a “sports technology partner.”

We analyzed each Big Tech company's presence and activities in the soccer media rights market by evaluating their activities concerning media rights (vertical axis) and their roles (sub-categories), similar to the approach used by Durrieu and Valette-Florence (57). The resulting diagrams display the general distribution of each company's activities in soccer (in %) and the intensities of these activities. The number of activities in each sub-category segment indicates the sub-category's contribution to a company's activities in professional soccer, and the intensity of shading indicates a company's presence in each sub-category, measured in terms of the number of activities. To estimate each company's presence, we count the number of activities undertaken by Big Tech companies in soccer rather than the financial value of contracts, as financial information is often unavailable.

It is noteworthy that there are similarities between the diagrams for Google and Facebook and Microsoft and Apple. These similarities, whether due to mimicry, innovation, competition, or the pursuit of new markets, raise questions and may indicate rivalries among Big Tech companies. Furthermore, Figure 5 illustrates that the potential for expansion in the media rights category (upper left-hand quadrant) is limited compared to the other areas. One could question whether companies still primarily acquire media rights to attract traffic. A deeper analysis of the Big Tech companies' financial investments would provide insight into each company's strategy. Finally, as shown in Figure 5, the Big Tech companies are currently not involved in the connected stadiums and fan engagement app markets (lower right-hand quadrant), even though this market has attracted other international IT companies such as IBM, Siemens, and HPE, the IT partner for Tottenham Hotspurs' new stadium (58). Cisco, a leading industry player, worked on the connectivity of Real Madrid's new Santiago Bernabeu stadium (59).

By examining the different actors and models of commercialization and distribution of media products, this study can serve as a starting point for understanding the transformation of the media landscape. One of the critical areas of focus for this study is the future impact of Big Tech in sports and soccer. As technology progresses, Big Tech companies are increasingly immersing themselves in the media sector, necessitating a thorough understanding of how their involvement will shape the future of sports and soccer media. This exploration includes assessing the influence of Big Tech firms on the distribution and consumption of media content, as well as the potential implications of emerging technologies such as virtual reality and augmented reality.

Another important aspect of this study is the evolution of actors through globalization. As digital media advances and global interconnectedness intensifies, it is imperative to comprehend how these actors adapt to cater to the shifting needs of a worldwide audience, for example, by investigating the roles of traditional broadcasters alongside emerging contenders such as social media platforms and streaming services. Finally, this study seeks to define the stakes of commercialization and distribution models of media products in connection with consumption patterns. As media consumption habits continue to evolve, it is crucial to understand how different commercial and distribution models shape how media products are consumed, for example, by examining the impact of subscription-based services and the role of advertising and sponsorships in the media industry.

## 5. Conclusion and discussion

Big Tech companies are relatively new players in the media rights market, but some have been involved in soccer since the mid-2000s. Like traditional media companies, Big Tech firms gain access to the soccer industry through investments in parallel markets, such as shareholdings and sponsorship, and sub-sectors linked to soccer, such as e-commerce, entertainment, and technological solutions. They aim to control the entire media rights sector, from production to distribution.

This exploratory study, which aims to provide an understanding of the involvement of Big Tech companies in soccer, identifies three groups of companies based on their activities in soccer: those active in the media rights market (Google, Facebook, Amazon), those interested only in non-live media rights (Netflix), and technology partners that are inactive in the media rights market (Apple, Microsoft). The results show that while acquiring media rights is a component of Big Tech's involvement in soccer, it is still a relatively minor aspect. Their main goal is not to become sports broadcasters but to use soccer to drive traffic to other services within their economic models. Big Tech companies employ a “glocal” approach in their development strategies, seeking to globalize their presence while also adapting to local conditions.

Our research indicates that Big Tech companies consistently collaborate with conventional broadcasters to acquire soccer media rights. The significance of these alliances between Big Tech enterprises and traditional broadcasters is profound. Early fears that Big Tech giants, such as Google and Facebook, might dismantle the traditional media sector and emerge as predominant forces have been largely dispelled. Instead, they are establishing collaborations with traditional broadcasters, recognizing them as complementary entities rather than replacements. These collaborations offer traditional broadcasters the opportunity to utilize the extensive reach and resources of Big Tech companies. This is particularly beneficial for smaller broadcasters, who may not possess the resource base or infrastructure of larger corporations. These broadcasters can harness their proficiency in online distribution, data analytics, and audience engagement by allying with Big Tech entities. This approach, in turn, allows them to reach a broader audience and maintain competitiveness in an evolving media landscape.

Simultaneously, Big Tech companies can benefit from these collaborations by gaining access to premium content and well-established audiences. Collaborating with traditional broadcasters provides them with expertise and experience, ensuring their platforms stay pertinent and engaging for users. Ultimately, the crux of the relationship between Big Tech firms and traditional broadcasters is their complementary nature. Despite Big Tech companies shaking up the traditional media industry, they also recognize the benefits of collaborating with established entities to unlock new opportunities and better cater to their audiences. This trend benefits the media industry, fostering greater innovation, cooperation, and expansion.

Furthermore, soccer serves as a large-scale test case for Big Tech companies to experiment with media rights markets and improve their algorithms, which can be applied to other industries. Interestingly, the COVID-19 pandemic provided an opportunity for Big Tech companies as online media consumption surged during lockdowns. Still, the lack of live content led some traditional media to sell rights at discounted prices. While Big Tech companies do not yet dominate the media rights market, they have established a strong presence in the markets they have entered. Traditional media will need to adapt quickly to succeed in this new landscape. Big Tech's economies of scope and scale will likely bring new ways of consuming soccer.

Premium rights continue to generate significant interest, but Big Tech companies also seem to place a high value on non-live content, which is a departure from traditional media companies' tendency to prioritize live content. However, the distribution model for live rights presents a significant challenge: Can pay-per-view and subscription-based formats be combined and made more widely accessible? Could a freemium hybrid content model align with the economic models of Big Tech companies? Additionally, integrating media rights with the videogame, betting, statistics, retail sales, and social network markets through the gamification of spectator sports could create a single product and significantly impact the entertainment industry.

The illegal broadcasting of Serie A matches on Facebook in 2016 and the issues with Amazon's broadcasting in 2019 and 2021 raise questions about the effectiveness of selling rights to Big Tech companies in protecting against illegal streaming. These companies may face challenges in safeguarding sports rights and ensuring the quality of live broadcasts. When Amazon acquired the rights to broadcast Premier League matches in 2019 and 2021, the promise of a new age of sports streaming was exciting to many. However, the reality has been somewhat challenging, and the difficulties have been multifaceted. Firstly, the issue of latency is significant in live sports streaming. Any delay between the live event and the broadcast can seriously affect the viewing experience, especially in the age of real-time social media updates. In Amazon's case, there were instances where the live stream lagged behind television broadcasts and even radio commentary. This latency issue can spoil the suspense of live matches, as viewers may learn about goals or fouls from other sources before they see them on the stream. Secondly, buffering issues have been reported by many users. Despite having robust server networks, Amazon has sometimes struggled to deliver a seamless streaming experience. During peak viewing times, such as high-profile matches, network congestion can cause the stream to buffer or reduce in quality. This is particularly problematic for viewers paying specifically for high-quality, uninterrupted access to these matches. The third issue is the challenge of protecting against illegal streaming. Despite efforts to secure their streams, Big Tech companies like Amazon have found it increasingly difficult to prevent unauthorized broadcasts of their content. The rise of sophisticated illegal streaming sites and the use of VPNs to bypass geographical restrictions means that the company's content can be accessed and shared illegally, often with impunity. Finally, there is the issue of providing a quality user experience across various devices. Viewers expect to be able to stream Premier League matches on their smart TVs, laptops, tablets, and smartphones. Ensuring compatibility and high-quality streaming across all these platforms can be a complex and costly process, and failures can lead to significant customer dissatisfaction.

In conclusion, this research offers vital preliminary insights into the relationship between Big Tech companies and the soccer industry, but it is not without its limitations. The collected data from 459 sources demonstrate that Amazon, Facebook, and Google accounted for 80% of the information, while the remaining 20% was associated with the other three companies. Though this study offers a snapshot of Big Tech's involvement with soccer, the disproportionate distribution of data may have skewed the results. Further research is warranted to comprehensively understand the financial investments of Big Tech companies in soccer and their strategies for commercializing and distributing media rights.

There are several potential research avenues that could deepen our understanding of the topics discussed. Investigating Big Tech's involvement in other sports, such as basketball, ice hockey, baseball, or American football, would offer a more comprehensive perspective. Incorporating Disney in future research is crucial, given its substantial investments in sports rights, such as the $2.6 billion Monday Night Football package and the projected $44.9 billion expenditure on sports rights through 2027. Moreover, our research did not address the financial aspects of contracts due to challenges in accessing data. Developing methods to incorporate this information would be a valuable contribution to future studies. Additionally, automating the data collection process could further advance knowledge by making data gathering more efficient, as manual transcription proved time-consuming in the current study. In summary, by exploring these avenues, researchers can significantly contribute to our understanding of Big Tech's involvement in the sports industry and help identify trends and opportunities in this rapidly evolving landscape.

## Data Availability

The original contributions presented in the study are included in the article, further inquiries can be directed to the corresponding author.
